# Compressed Air

**Published:** 1873-03

**Authors:** J. H. Etheridge

**Affiliations:** Prof. of General Therapeutics, Rush Medical College, Chicago


Article IV.—*Compressed Air.* Synopsis of a Lecture delivered before the Rush Medical College Students, Dec., 1872. By J. H. Etheridge, M.D., Prof. of General Therapeutics, Rush Medical College, Chicago.

*Gentlemen:*—The therapeutical advantages of this agent are being shown to us by a few German and French investigators, and I propose, to-day, to briefly lay before you the results of the labors of one of these gentlemen, Dr. J. Lange, of Dresden, who has kindly sent me his published pamphlets on the subject.

Passing over the preliminary considerations of the subject, I invite your attention to a description of the apparatus used in administering compressed air baths. Due regard must be had to ventilation, and to ability to measure precisely the atmospheric pressure in constructing the apparatus. To meet these ends, the so-called "air bath bells," are constructed of iron sheets welded together hermetically. The bells are six feet in diameter, and eight or nine feet high, provided with a door opening inwardly. In the centre of the floor is a supply tube, connected with the pumps; about four inches above this tube is a circular platform, nearly six feet in diameter, and upon this are placed the chairs for the patients to sit on. The patient takes a seat, the pumps are set in motion, and air is forced in gradually, almost inperceptibly diffusing itself throughout the bell, its outward pressure shutting the door hermetically, and the platform so distributing it—the air—as to avoid subjecting the individual to a draft.

At the top is a tube leading from the bell, which bifurcates—one arm being opened by a stop cock. Through this cock the air is allowed to pass during the whole bath—thus securing good ventilation. The pumps work constantly, and the pressure is regulated by the stop cock. When it is desired to gradually increase the amount of air-pressure, less is allowed egress through the waste pipe than comes in through the supply pipe under the platform, and *vice versa*. To the other arm of this pipe at the top of the bell, is attached a manometer, which indicates the air pressure within the bell with exactitude.

The bath generally lasts two hours—the first half hour is engaged in bringing the pressure gradually to the maximum—during the ensuing hour, the pressure is maintained without any variation, and during the last half hour the pressure is decreased till the normal standard is attained.

I now invite your attention to the physiological and therapeutical effects of this simplest of all remedial agents. They are really wonderful, and it will not be long before the facilities for administering compressed air baths will be found common enough in large towns.

### EFFECTS ON RESPIRATION.

During the bath, the inspiratory acts are notably easier, that is, the power of the inspiratory muscles seems to be increased. The respirations become longer and slower, and connected with them is a sensation of easiness. Both these effects are noticeable in the healthy, but especially are they noticed by those patients troubled with thoracic diseases.

### EFFECTS ON THE CHEMISTRY OF RESPIRATION.

Observations on this point are meagre. Up to a certain point of pressure the expiration of carbonic acid goes beyond the normal standard; at a higher pressure the amount of carbonic acid diminishes, and becomes less than in an ordinary atmosphere. An after effect of the bath consists in an augmented expiration of carbonic acid, which continues for some time subsequently, eventually attaining a maximum, and then decreasing to the normal standard.

### EFFECTS ON THE CIRCULATION.

To appreciate the remarkable effects of compressed air on the circulation, you must call to mind some of the forces constantly in play during life. *Outside* of the lungs, yet within the hermetically tight thoracic walls, are the heart and larger vessels. During ordinary *inspirations* these vessels are pressed upon by the outward distending lungs—the venous blood being impeded in its flow, and the arterial flow being promoted. This retarding the one, and promoting the other, is only *slight* in the ordinary atmosphere. During ordinary *expirations*, the mouths of the large veins opening into the thoracic cavity are made somewhat patulous, and this patulous condition produces aspiration, and the capillary blood is *sucked onward* towards the heart. Let me emphasize the fact, that this aspiration is only *slight* in degree—yet it is a small factor in ordinary circulation. In the compressed air bath, the effects of the air on the arterial and venous flow during *inspiration* and *expiration* are greatly increased, and we have, as a consequence, augmented *arterial force* (of flow), and *increased aspiration* at the mouths of the large veins, which aspiration results in an unusual activity and completeness of capillary circulation. Increased aspiration at the mouth of the subclavian causes a more complete evacuation of the thoracic duct.

In the bath, decreased heart pulsations are *never* absent in the healthy, and especially in patients with pulmonary troubles. The rate of decrease is not uniform in all individuals, nor at all times in the same person. At one time the diminution may be four or five beats—at another it may be twelve, fifteen or twenty beats. Bertin had a case of double emphysema, with the pulse at 106 or 108. After the first bath it fell to seventy-two beats—and continued to fall daily thereafter (under daily baths), till it reached forty-five, and there remained for a time, and for a long time thereafter it never went above fifty-six. In cases of lung trouble, where the pulse is too high, the baths always cause a diminution, which is permanent when the cure is established.

### EFFECTS ON THE ANIMAL HEAT.

The thermometer always indicates an increase of temperature. When the pressure is diminished the heat is decreased, and the patient becomes sensible of it. If the pressure is suddenly decreased, heat is suddenly made latent and the air vapor is precipitated in a thick fog. In many instances a disagreeable shivering is experienced in such trials.

### EFFECTS ON MUSCULAR POWER.

The power of not only the inspiratory muscles is increased, but that of the whole muscular system, as may be proven by conducting experiments with lifting heavy weights with the hand. Under increased pressure increased amounts of weight can be lifted.

### EFFECTS ON THE NERVE CENTRES.

In many persons is aroused a "feeling of spritual well-being, levity and liberty." One author, Tunod, asserts that it produces a flow "of rich ideas, with a tendency to verse making"!

### EFFECTS ON TISSUE METAMORPHOSIS.

1*st. Digestion.* The baths always produce invigoration of the digestive function, as is shown by the increased appetite and the greater action of the intestinal offices. Increased aspiration on the subclavian causes increased speed of flow of lymph from the thoracic ducts into it. The thoracic duct is greater pressed upon by inspiration, and the flow of lymph is thereby accelerated.

2*d. Urine.* In most cases the urine is increased in quantity. Sulphates are increased—phosphates continually decrease till they disappear.

3*d. Augmentation of Weight.* This result almost invariably follows the prolonged use of the baths. In one case the weight increased from 154 to 162 pounds in thirty-eight days. In another, from 134 to 144 pounds in twenty-one days.

### CONCLUSIONS.

All phenomena observed from compressed air-baths point to the one conclusion, that the nutrition is made more normal and better. All nutrition changes take place in the capillaries. The immediate effect of a bath is to induce capillary movement, such as many parts of certain systems seem to be strangers to, most of the time. The conditions seeming to call loudly for these baths are characterized by poverty of blood, deficient formation of blood, (as seen in chlorosis and in convalescents after debilitating diseases).

Special attention is called to the various thoracic diseases, as treated by this means, and results arising therefrom.

IN CATARRHAL DEAFNESS,

Unpleasant pressure on the tympani is experienced at first in the bath, but swallowing powerfully will equalize the air pressure on each side of the tympanum. Compressed air coming thus in contact with the congested eustachian mucous membrane, exerts a most happy effect on it, and the deafness disappears after a few baths.

IN LARYNGITIS,

Recent cases are almost invariably *speedily* cured by this treatment. Chronic cases are cured more slowly. Singers affected with laryngitis and hyperæmia of the vocal cords, sufficient to prevent vibration, and thus induce aphonia, are readily cured. Cures seem to remain thoroughly and persistently when treated with compressed air.

CHRONIC BRONCHITIS OR CATARRH.

In these cases we have hypertrophy and thickening of the mucous membrane and muscular coat, with a pus like, yellow or tenacious semi-transparent secretion, with loss of elasticity of the mucous membrane and muscular coat, and, therefore, much inclination to distention of the bronchi and to emphysema. Under compressed air baths the secretion is speedily checked, the mucous membrane and muscular coat regain their natural strength, and in most cases the amendment is so magical as to surprise not only the patient but the medical attendant. All these curative processes are effected solely through a bettered nutrition. There is not much hope of cure of sacculated dilatations in this trouble.

IN CONVALESCENCE

From acute bronchitis — especially the capillary form — compressed air is particularly applicable, for two reasons:

1st. To remove the residue of the disease, and the beginnings of sequelæ, as distension and emphysema.

2d. To secure prophylaxis against relapses, which are a prominent feature of after conditions, from capillary bronchitis and ordinary bronchitis.

IN DISEASES OF LUNG PARENCHYMA,

The effects of this agent are astonishing, but especially noticeable are its good results in emphysema. Until observations were made with this remedial agent, idiopathic emphysema was deemed incurable; now, however, we see the patient running up stairs, or ascending mountains, who was once unable to take a few steps on a level without gasping for breath—sleeping in any position at night, when previously the horizontal position would have been death to him; we hear normal sounds in the lung; we find the liver and heart have found their normal position; we find a great diminution in chest measurements, and a disappearance of the "barrel-shaped" chest.

All these changes are effected in a few weeks by baths—changes which no other agent has ever secured in emphysema previously.

TUBERCULOSIS OF THE LUNG.

Deficient nutrition causes tubercle in properly disposed persons. Hyperoxidation restores nutrition in such cases. Under compressed air baths all the bad symptoms of this disease slowly disappear. The spirometer actually shows increased lung capacity. Tubercles become atrophied and calcify, and the soft matters of them become absorbed. Even where vomicæ exist, much good is done by baths. Where interstitial tubercularization exists, no hope can be entertained.

I hoped to have had time to allude to the sparse experiments made in cases of organic heart disease, but I cannot do it at this time. I trust I have said sufficient to convince you that we have an unequaled agent in compressed air, and I hope that the day is not far distant when the baths can be taken in any town of our country, and will be freely used by rational, intelligent physicians.

